# Autophagy and Exosomes: Cross-Regulated Pathways Playing Major Roles in Hepatic Stellate Cells Activation and Liver Fibrosis

**DOI:** 10.3389/fphys.2021.801340

**Published:** 2022-02-03

**Authors:** Eleftheria M. Mastoridou, Anna C. Goussia, Georgios K. Glantzounis, Panagiotis Kanavaros, Antonia V. Charchanti

**Affiliations:** ^1^Department of Anatomy-Histology-Embryology, Faculty of Medicine, School of Health Sciences, University of Ioannina, Ioannina, Greece; ^2^Department of Pathology, Faculty of Medicine, School of Health Sciences, University of Ioannina, Ioannina, Greece; ^3^Hepato-Pancreatico-Biliary Unit, Department of Surgery, University General Hospital of Ioannina and School of Medicine, University of Ioannina, Ioannina, Greece

**Keywords:** autophagy, exosomes, hepatic stellate cells, liver, fibrosis

## Abstract

Chronic liver injury, regardless of the underlying disease, results in gradual alteration of the physiological hepatic architecture and in excessive production of extracellular matrix, eventually leading to cirrhosis Liver cellular architecture consists of different cell populations, among which hepatic stellate cells (HSCs) have been found to play a major role in the fibrotic process. Under normal conditions, HSCs serve as the main storage site for vitamin A, however, pathological stimuli lead to their transdifferentiation into myofibroblast cells, with autophagy being the key regulator of their activation, through lipophagy of their lipid droplets. Nevertheless, the role of autophagy in liver fibrosis is multifaceted, as increased autophagic levels have been associated with alleviation of the fibrotic process. In addition, it has been found that HSCs receive paracrine stimuli from neighboring cells, such as injured hepatocytes, Kupffer cells, sinusoidal endothelial cells, which promote liver fibrosis. These stimuli have been found to be transmitted *via* exosomes, which are incorporated by HSCs and can either be degraded through lysosomes or be secreted back into the extracellular space *via* fusion with the plasma membrane. Furthermore, it has been demonstrated that autophagy and exosomes may be concomitantly or reciprocally regulated, depending on the cellular conditions. Given that increased levels of autophagy are required to activate HSCs, it is important to investigate whether autophagy levels decrease at later stages of hepatic stellate cell activation, leading to increased release of exosomes and further propagation of hepatic fibrosis.

## Introduction

Liver fibrosis is a homeostasis disorder defined by increased synthesis and deposition of extracellular matrix (ECM) and a parallel decrease of physiological mechanisms underlying matrix degradation or remodeling (Lotersztajn et al., 2005; Mallat and Lotersztajn, 2013). Liver is considered a complex tissue, consisting of different cell populations, however hepatic stellate cells (HSCs) have been demonstrated to play a major role in liver fibrosis (Geerts, 2001).

Fibrosis progression results from excessive ECM deposition, predominantly produced by activated hepatic stellate cells (aHSCs), and failure of matrix degradation (Iredale et al., 2013). In the normal liver, quiescent hepatic stellate cells (qHSCs) reside in the perisinusoidal space (space of Disse), interposed between hepatocytes and liver sinusoidal endothelial cells (LSECs), displaying a quiescent phenotype, characterized by the presence of retinyl esters. Upon chronic liver injury, qHSCs become activated and they eventually acquire a myofibroblast-like phenotype, which leads to increased production of ECM (Lotersztajn et al., 2005). So far, it is well known that autophagy is a key component of HSCs activation process, as it has been demonstrated to be implicated in their phenotypic alterations through digestion of their stored lipid droplets (Hernández-Gea et al., 2012). Moreover, exosomes, a type of extracellular vesicles (EVs), have recently become of great interest as they are implicated into the pathogenesis of liver fibrosis by contributing to HSCs activation and migration to sites of fibrogenesis (Huebert et al., 2010; Charrier et al., 2014). This review highlights the multifaceted role of autophagy in HSCs activation, and the recent advances on the molecular and vesicular interaction between autophagic pathway and exosomes considering HSCs activation during liver fibrosis.

### Mechanisms of Hepatic Stellate Cells Activation During Liver Fibrosis

Liver fibrosis is a prolonged wound healing process characterized by distorted hepatic architecture and excessive ECM accumulation, produced by a heterogeneous population of myofibroblasts, such as HSCs, bone-marrow derived fibrocytes, portal myofibroblasts and epithelial to mesenchymal transition (Mederacke et al., 2013). Myofibroblastic cells migrate and accumulate at sites of liver parenchymal injury in response to various factors derived from neighboring injured cells and from extensive changes in ECM composition (Lotersztajn et al., 2005; Mallat and Lotersztajn, 2013). Despite the fact that HSCs in the normal liver represent 5–8% of the total number of liver cells (Geerts, 2001), they constitute the main source of liver fibrogenic cells, contributing 82–96% of myofibroblasts (Mederacke et al., 2013).

In the homeostatic liver, HSCs are “quiescent” (qHSCs) and have a starlike configuration due to their dendritic cytoplasmic extensions, which let them interact with hepatocytes, adjacent HSCs and LSECs (Gressner and Weiskirchen, 2006) and are characterized by the expression of a large panel of adipogenic genes, such as peroxisome proliferator-activated receptor gamma (PPARγ) (Kisseleva et al., 2012) and neural/neuroendocrine markers, such as glial fibrillary acid protein (GFAP) (Morini et al., 2005), nestin, neurotrophin receptor, and synaptophysin (Eng and Friedman, 2000). A characteristic feature of qHSCs is the presence of large perinuclear lipid droplets, which serve as the main storage site for vitamin A and are essential in the regulation of retinoic acid homeostasis (Carpino et al., 2004). Moreover, qHSCs play a crucial role in several other specialized functions in the normal liver, including ECM remodeling and homeostasis (Geerts, 2001) by secretion of ECM components and matrix metalloproteinases (MMPs), and control of sinusoidal blood flow *via* contraction or dilation of the sinusoidal lumen (Semela et al., 2008). Moreover, qHSCs are implicated in the secretion of growth factors and cytokines such as hepatic growth factor (HGF), vascular endothelial growth factor (VEGF), and insulin growth factor (IGF) (Friedman, 2008a). These secretory functions that contribute to intercellular communication between adjacent HSCs, LSECs, Kupffer cells (KCs) and hepatocytes are potentially mediated through a type of extracellular vesicles named as “exosomes” (Masyuk et al., 2013; Sung et al., 2018). The role of exosomes in HSCs activation and liver fibrosis will be discussed thoroughly below.

As a result of disturbances in hepatic homeostasis after liver injury, qHSCs undergo “activation” or transdifferentiation, from a quiescent, vitamin A –storing cell to a myofibroblast-like cell, with the loss of lipid droplets being a hallmark of HSCs activation. During activation, qHSCs acquire several new phenotypic characteristics, such as expression of α-Smooth muscle actin (αSMA), fibrillary collagens (mainly type I and type III), increased expression of tissue inhibitors of metalloproteinases (TIMPs), platelet-derived growth factor receptor beta (PDFGR-β), transforming growth factor beta (TGF-β) and endothelin-1 (ET-1) (Higashi et al., 2017; Parola and Pinzani, 2019). Consequently, aHSCs exhibit profibrogenic and proinflammatory properties, as they produce chemokines (e.g., interleukin 6 (IL-6) capable of recruiting inflammatory cells and other HSCs and they are characterized by increased proliferation rate, contractility, motility and synthesis of ECM components (Friedman, 2008a).

Activation of HSCs consists of two phases; initiation and perpetuation (Friedman, 2000). Initiation or “preinflammatory” phase encompasses paracrine stimuli derived from injured hepatocytes, neighboring LSECs and KCs and changes in ECM composition, which result in early changes in gene expression in HSCs with aim to render them more responsive to further paracrine stimulation. The second phase, termed as perpetuation incorporates cellular events that amplify the activated phenotype of HSCs. Increased expression of cell membrane receptors results in enhanced responsiveness to cytokines and growth factors (Friedman, 2000). Interaction of aHSCs with fibril forming ECM, mainly by membrane adhesion receptors such as integrins, accelerates their activation and modulates further the function of several key profibrotic pathways (Yang and Klionsky, 2009; Mijaljica et al., 2012). The main phenotypic responses of aHSCs during perpetuation include proliferation, contractility, fibrogenesis, dysregulation of matrix degradation, chemotaxis, retinoid loss and cytokine release (Lee and Friedman, 2011; Tsuchida and Friedman, 2017).

During initiation, various signals (reactive oxygen species-ROS, VEGF, PDGFβ, TGFβ) from damaged neighboring cells, such as apoptotic hepatocytes, KCs, LSECs and platelets, as well as changes in the surrounding extracellular matrix, result in genetic and transcriptional modifications, rendering HSCs more responsive to cytokines and stimuli. Induction of the expression of PDGFR-β in aHSCs is a hallmark of early activation and is followed by development of contractile and fibrogenic phenotype (Friedman, 2008b; Lee and Friedman, 2011). Moreover injury stimuli induce signaling pathways, which result in the degradation of lipid droplets through lipophagy (Lee and Friedman, 2011). Maintenance of the activated phenotype triggers the second phase of the activation program (perpetuation) and the acquisition of fibrogenic and proinflammatory properties (Lotersztajn et al., 2005; Mallat and Lotersztajn, 2013). Upon activation, aHSCs continue to receive paracrine stimuli, but they simultaneously synthesize and secrete soluble factors acting in an autocrine fashion such as TGF-β, PDGF-β, and ET-1 (Li et al., 2008; Lee and Friedman, 2011). *In vitro* cultures of HSCs have demonstrated morphological differences between initiation and perpetuation HSCs, including the evidently higher levels of a-SMA protein expressed in perpetuation HSCs. Moreover, the same study has shown that molecules derived by initiation HSCs seem to protect hepatocytes form cell death, while on the other hand perpetuation HSCs exhibited active synthesis and secretion of fibrogenic molecules (Chang et al., 2015), secreted mainly *via* exosomes and affecting both other aHSCs and qHSCs, thus amplifying the fibrogenic process (Charrier et al., 2014).

Resolution of the injury stimuli may result in attenuation or even regression of liver fibrosis by inducing cell death, senescence or regression of aHSCs to their quiescent phenotype (Campana and Iredale, 2017).

### Autophagy in Hepatic Stellate Cells and Liver Fibrosis

#### Overview of Autophagy

Autophagy is an evolutionarily conserved catabolic process in which intracellular macromolecules and organelles (referred to as “autophagic cargo”) undergo lysosomal degradation. There are 3 main types of autophagy: macroautophagy (thereby referred to as “autophagy”), microautophagy and chaperone-mediated autophagy, all of which differ in their delivery methods to the lysosome (Mizushima and Komatsu, 2011). Autophagy occurs constitutively (basal or constitutive autophagy), but is substantially enhanced under stress conditions, such as nutrient or energy starvation (stress-induced autophagy) (Parzych and Klionsky, 2014). Normal autophagy is crucial for maintenance of liver homeostasis, whereas autophagy dysfunction is associated with pathological conditions (Wirawan et al., 2012). Mounting evidence indicates that dysregulation of the autophagic flux in parenchymal and non-parenchymal liver cells (HSCs, LSECs, KCs) drive the progression of various liver diseases.

Measuring the autophagic flux is of great interest since autophagic levels affect the function and the structure of the cell and the whole tissue. The cytosolic form of MAP1LC3/LC3 (microtubule-associated protein 1 light chain 3), termed LC3-I, is lipidated to form LC3–phosphatidylethanolamine, LC3-II, which is specifically recruited to the phagophore membrane. LC3-II, present in the inner membrane of the autophagosome remains conjugated during the autophagosome maturation, thus levels of LC3-II correlate well with the autophagic flux (Loos et al., 2014). Degradation of p62 is another widely used autophagic marker due to the fact that p62 directly binds to LC3-II and is selectively degraded within the autophagosome (Pankiv et al., 2007). Levels of autophagy must be strictly regulated, as both excessive and insufficient autophagic flux have been associated with disruption of liver homeostasis and genesis of various liver diseases (Rautou et al., 2010; Codogno and Meijer, 2013; Czaja et al., 2013). With regard to liver fibrosis, autophagy has emerged as a complex regulator, both with profibrogenic and antifibrogenic properties depending on the liver cell-type (Mallat and Lotersztajn, 2013).

The main molecular signaling pathway by which autophagy exerts its multifunctional role during liver fibrosis is through Phosphoinositide 3-kinase/Protein kinase B (PKB), also known as Akt/mammalian Target of Rapamycin (PI3K/Akt/mTOR) (reviewed in Wang H. et al., 2019). mTOR consists of two distinct signaling complexes mTORC1 and mTORC2. PI3Ks, members of the intracellular lipid kinase family, produce phosphoinositides that act as a second messenger that recruits Akt (Sarbassov et al., 2005), a serine/threonine protein kinase (Morales-Ruiz et al., 2017). mTORC1, which is the main endogenous autophagy inhibitor, is then activated by Akt mainly *via* direct phosphorylation and inactivation of Tuberous sclerosis complex (TSC) (inhibitor of mTORC1).

Basal or constitutive autophagy, which occurs under nutrient rich conditions and is mainly implicated with the quality control of intracellular proteins and organelles, is insensitive to inhibitory effects of mTOR, since mTOR signaling pathway is mainly associated with stress induced autophagy. Nutrient starvation, stress conditions and reduced availability of growth factors suppress mTOR signaling pathway, thus allowing initiation of autophagy. However, autophagy has a role in the unstressed liver, implicated in the elimination of damaged organelles and altered proteins even under nutrient rich conditions. Therefore, autophagy not only plays a principal role in the supply of nutrients for liver cell survival, but also plays a constitutive role in cellular homeostasis by acting as a cytoplasmic quality control mechanism (Al-Bari and Xu, 2020).

Although there are discrepancies regarding the role of autophagy as a promoter or inhibitor of liver fibrosis, there are several lines of evidence that PI3K/Akt/mTOR pathway is a major molecular mechanism that is regulated from upstream signals such as growth factors, energy status, oxygen levels (Saxton and Sabatini, 2017). However, under energy-deprivation conditions in qHSCs, mTOR signaling pathway is inhibited and thus autophagy could be initiated to produce ATP levels and confront enhanced energy demands upon myofibroblast transformation (Huang et al., 2019). On the contrary, mounting evidence suggests that increased autophagy levels *via* inhibition of mTOR signaling pathway have been associated with reduction of HSCs activation and alleviation of liver fibrosis (Lee et al., 2014; Zhang et al., 2018).

#### Role of Autophagy in Hepatic Stellate Cells Activation and Liver Fibrosis

Liver fibrosis is characterized by excessive ECM production, with HSCs regarded as the major cellular source of collagen producing myofibroblasts. Activation of HSCs and transformation into a fibrogenic phenotype is a hallmark in the propagation of hepatic fibrosis. HSCs activation is a dynamic process that highly depends on autophagy, a prominent pathway, the levels of which are closely regulated by the cellular microenvironment (Weiskirchen and Tacke, 2016).

Transformation of HSCs from the quiescent to the activated phenotype consists of two phases; initiation and perpetuation, as mentioned above. Autophagy plays a key role in both phases. During initiation, dysregulation of the autophagic flux in neighboring cell populations such as injured hepatocytes, KCs and LSECs results in loss of homeostasis and release of proinflammatory signals and profibrogenic cytokines affecting qHSCs. More specifically, the quiescent phenotype of HSCs has been demonstrated to be maintained by normal LSECs (Poisson et al., 2017), as LSECs are the first cell population to sense injury stimuli due to their position (Deleve et al., 2008). However, chronic liver injury results in downregulation of the autophagic levels in LSECs, thus their inability to handle oxidative stress and the endothelial inflammation along with significant phenotypic alterations, such as capillarization of LSECs, have been associated with activation of qHSCs (Ruart et al., 2019). During the initiation phase, paracrine stimuli transferred between injured cells and qHSCs, mainly through exosomes, result in morphological changes and genetic expression of various proteins in HSCs (Sung et al., 2018). This “priming” phase, which is evidently associated with dysregulation of the autophagic flux in adjacent to HSCs liver cell types is a prerequisite for the HSCs to become more responsive to the paracrine signals and enter the perpetuation phase (Ezhilarasan, 2020).

Finally, the main feature of the initiation phase in HSCs is the loss of retinyl ester-containing lipid droplets and of adipogenic factors. Autophagy has been demonstrated to play a crucial role in this process through digestion of lipid droplets by selective autophagy known as lipophagy (Thoen et al., 2011; Hernández-Gea et al., 2012), thus determining the activated phenotype of HSCs and the acquisition of contractile, proliferative, inflammatory, and fibrogenic properties (Friedman, 2008a). Autophagy provides energy that is essential to support HSCs activation through lipid droplets mobilization, liberation of free fatty acids, and mitochondrial β-oxidation. Energy deprivation along with stressful stimuli in qHSCs inhibit mTOR signaling pathway, which is the main endogenous regulator of the autophagy during liver fibrosis (Wang H. et al., 2019). On the other hand, autophagy-deficient qHSCs may be unable to degrade lipid droplets (LDs), resulting in decreased free fatty acids (FFA) availability and ATP production (Hernandez-Gea and Friedman, 2011). The elevated autophagic levels during initiation phase have been confirmed by an increase in the autophagic marker LC3-II and a decrease in p62 expression upon HSCs activation, indicative of enhanced autophagic flux (Hernandez-Gea and Friedman, 2011).

Thus, HSCs activation is a sequential process that does not only require the autophagy of their lipid droplets. Before the induction of lipophagy, HSCs have already interacted with their neighboring cells by responding to autocrine signals and paracrine secreted inflammatory cytokines, growth factors and miRNAs, many of which are transferred *via* exosomes (Wang et al., 2015). However, it is necessary to underline the fact that autophagy is demonstrated as the main regulator both in the secretion of inflammatory cytokines from LSECs and KCs, which contribute to the initial interruption of the quiescent phenotype of HSCs, as well as in the final activation of HSCs by the autophagic degradation of their lipid droplets.

On the contrary, during the perpetuation phase, autophagy is downregulated. mTOR signaling pathway is also the main regulation during this phase, as its activation from various intercellular growth factors (i.e., PDGF) inhibits autophagy (Gao et al., 2020). The impairment of the autophagic flux is also reflected by the aggregation of p62, despite the upregulated levels of LC3-II (Zhang et al., 2020). Decreased levels of autophagy during perpetuation are necessary for the progression of the fibrosis as external stimulation of autophagy has been associated with attenuation of liver fibrosis (Lucantoni et al., 2021).

Although numerous studies have demonstrated the significance of the autophagic process in HSCs activation and hepatic fibrosis, there are some discrepancies regarding its role as a profibrogenic or antifibrogenic factor. Various experiments have been conducted both *in vivo* and *in vitro* to further investigate the role of autophagy in HSCs activation and transformation into a myofibroblast like cell (Lucantoni et al., 2021).

Several reports are consistent with the first described study in 2012 (Hernández-Gea et al., 2012), as they have demonstrated that increased autophagy is not only concomitant, but it is essential for HSCs activation. Experiments in *in vitro* cultures of HSCs or primary cell lines have confirmed the profibrogenic role of autophagy as it is upregulated upon activation of HSCs (Deng et al., 2014; Chen M. et al., 2017; Hong et al., 2018; Lin et al., 2018). Of note, data from primary human cell lines and cell cultures from patients are rare, but in accordance with the fact that autophagy is essential for HSCs activation (Thoen et al., 2011; Hernández-Gea et al., 2012; Kim et al., 2018). In *in vivo* experiments, the examination of whole fibrotic tissues has shown increased autophagosome formation (Hernández-Gea et al., 2012; Deng et al., 2014; He et al., 2014; Kong et al., 2019; Wang Y. et al., 2019), without, however, investigating solely HSCs and how the autophagic flux is affected during the different stages of their activation. Initiation phase seems to be regulated by enhanced autophagy, both indirectly by secretion of proinflammatory cytokines from neighboring liver cells, due to dysregulation of their autophagic levels, that eventually disrupt the quiescent phenotype of HSCs, and directly by inducing degradation of lipid droplets in HSCs to confront increased energy demands during their myofibroblast like transformation.

On the other hand, it is essential to be mentioned that there are studies that are not in keeping with the concept that autophagy promotes HSCs activation and liver fibrosis. On the contrary, it has been demonstrated that induction of autophagy has been associated with inhibition of HSCs activation and antifibrotic effects.

**FIGURE 1:**
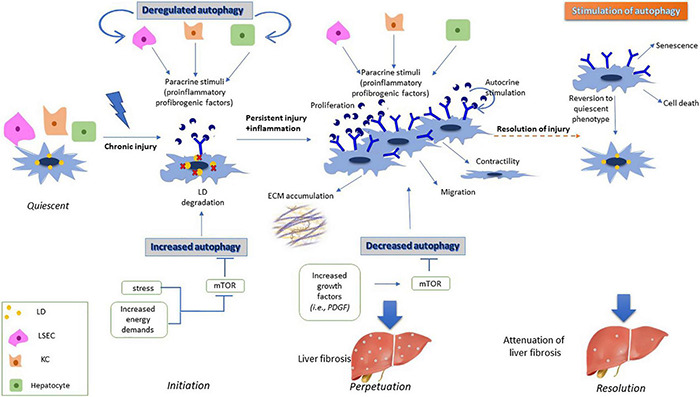
Effects of autophagy on HSC activation. During initiation phase, dysregulation of autophagy in injured liver cells such as LSECS, KCs and hepatocytes result in secretion of proinflammatory and profibrogenic signals. These signals are incorporated by HSCs, thus inducing expression of various membrane receptors, and becoming more responsive to external signals. Moreover, increased energy demands of HSCs during their transformation to myofibroblast cells results in degradation of their lipid droplets through lipophagy. During perpetuation phase, increased expression of growth factors in aHSCs leads to downregulation of autophagy, with aHSCs having acquired fibrogenic properties such as proliferation, contractility and secretion of ECM components, thus leading to liver fibrosis. However, induced autophagy during the perpetuation phase leads to either senescence, cell death or reversion of aHSCs to their quiescent phenotype resulting in attenuation of the fibrotic process.

*In vitro* experiments have shown that natural compounds, such as Oroxylin A, a safe natural product with well-established anti-inflammatory and anti-oxidant properties, and caffeic acid phenethyl ester, a phenolic compound with strong biological properties in liver protection, used in primary cell lines of aHSCs, exert their antifibrotic role as autophagy inducers, while they have been also associated with inhibition of HSCs activation (Yang et al., 2017; Chen et al., 2018). Autophagy may act as an anti-fibrotic factor, *via* induction of cell death in aHSCs, thus inhibiting their proliferation (Piguet et al., 2014; Zhang et al., 2018). Moreover, the antifibrotic effects of autophagy have been associated with the downregulation or degradation of profibrogenic factors in aHSCs, such as collagen and exosomes (Seo et al., 2014). These findings have also been confirmed in animal models, but without information from human liver tissue, thus contributing to attenuation of hepatic fibrosis both *in vitro* and *in vivo* (Piguet et al., 2014; Chen et al., 2018; Gao et al., 2020; Zhang et al., 2020). These experiments have been conducted in fibrotic liver tissues, meaning that HSCs had already been in the perpetuation phase. Autophagic levels are downregulated in the perpetuation phase, thus leading to increased secretion of EVs and contributing to the propagation of liver fibrosis (Gao et al., 2020). However, external overstimulation of autophagy in this phase has shown attenuation of liver fibrosis through induction of cell death, senescence or downregulation of all the fibrogenic features of the already activated HSCs (reviewed by Lucantoni et al., 2021). The multidimensional role of autophagy during HSCs activation is displayed in Figure 1.

### Extracellular Vesicles and Liver Fibrosis-Role in Hepatic Stellate Cells Activation and Migration

#### Overview of Extracellular Vesicles

EVs are lipid bilayer membrane structures secreted into the extracellular space by normal, diseased and transformed cells, which play an important role in cell to-cell communication both basally and under pathological conditions (Gould and Raposo, 2013; Kowal et al., 2014). Liver is a functionally complex organ, exhibiting numerous different properties and thus, intercellular communication and exchange of information is crucial for the maintenance of liver homeostasis (Masyuk et al., 2013). Thus, EVs have become of great interest because of their important role in intercellular communication between liver cells (Masyuk et al., 2013; Hirsova et al., 2016). Furthermore, EVs were first described as a means of elimination of unneeded cellular compounds, which up to date is considered as a secondary function. They consist of two major size categories, namely plasma membrane derived microvesicles (50–1,000 nm) and endosome-derived exosomes (30–150 nm in diameter) as defined by the International Society for Extracellular Vesicles” (ISEV) and according to the Minimal Information for Studies of Extracellular Vesicles (MISEV) guidelines of 2014 (Lötvall et al., 2014). Most liver cell types such as hepatocytes, LSECs, HSCs, KCs are exosome secreting cells and exosome responding cells (Deng et al., 2017), thus exosomes will be the focus of our study. Although all liver cell types secrete different exosome cargoes under pathological conditions, HSCs derived exosomes are considered as the most prominent indicator of the extent of liver injury (Sung et al., 2018).

Exosomes contain a variety of cargoes including lipids, proteins, mRNAs, micro-RNAs (miRNAs) and long-non-coding RNAs (lncRNAs) and they can be transferred from donor cells to recipient cells, where they can activate or regulate cell activities such as protein expression, cell proliferation and differentiation or metabolic alterations (Vlassov et al., 2012). Exosome proteins can be classified as non-specific, which are mainly associated with their biogenesis pathway, and as specific, which reflect the pathophysiological state of the donor cell (Denzer et al., 2000). Both the quantity and the quality of the secreted exosomes vary under diverse conditions, depending on the parental cells (Cai et al., 2017). The cell-type specific cargoes of exosomes have a different impact on the surrounding recipient cells, by inducing genetic or phenotypic alterations, thereby contributing to maintenance of homeostasis or to propagation of diseases (Povero et al., 2015; Ban et al., 2016).

#### Biogenesis of Exosomes

Exosomes are spherical to cup-shaped membranous vesicles with a size of 30–100 nm. They are formed by inward protrusions of the limiting membrane of early endosomes as small intraluminal vesicles (ILVs), which mature into late endosomes/multivesicular bodies (MVBs) (Raposo and Stoorvogel, 2013; Yáñez-Mó et al., 2015; Bebelman et al., 2018). MVBs may follow either degradative pathways or fuse with the plasma membrane and release their ILVs into the extracellular lumen, which are now termed as “exosomes” (Simons and Raposo, 2009; Bebelman et al., 2018).

Exosome biogenesis is associated with two distinct trafficking pathways; ESCRT (endosomal sorting complex required for transport)- dependent and ESCRT independent pathways (Hurley and Odorizzi, 2012). There are four different ESCRT complexes, each one involved with distinct functions during exosome biogenesis. Several ESCRT proteins are included in the four complexes, such as hepatocyte growth factor-regulated tyrosine kinase substrate (HRS) (Razi and Futter, 2006; Tamai et al., 2010), tumor susceptibility gene 101 protein (TSG101) (Razi and Futter, 2006), which are involved in the initial stages of exosome biogenesis, apoptosis-linked gene-2 interacting protein X (Alix), which has been shown to promote intraluminal budding of vesicles in early endosomes (Baietti et al., 2012) and vacuolar protein sorting-associated protein 4 (VPS4), which is associated with the final steps of ILV formation, membrane scission and dissociation of ESCRT complexes (Babst et al., 2011).

ESCRT independent pathways involve exosome biogenesis in the absence of the ESCRT protein machinery (Stuffers et al., 2009). Ceramides are the most studied lipid driven mechanism involved in the formation of exosomes. Ceramides are associated with the formation of microdomains into ILVs and the inward curvature of the limiting membrane of ILVs to form MVBs (Trajkovic et al., 2008). Furthermore, tetraspanins (CD9, CD10, CD26, CD63, CD81, CD82) (van Niel et al., 2018), which are transmembrane proteins, are implicated in exosome formation in an ESCRT-independent manner. Tetraspanins are involved in the formation and cargo sorting in ILVs (van Niel et al., 2011; Perez-Hernandez et al., 2013) and in exosome release (Hurwitz et al., 2016).

The fate of MVBs is either the degradation pathway, mediated by fusing with the lysosome compartment or the secretion pathway, with MVBs being released as exosomes. Exosome release appears to be controlled by RAB guanosine triphosphates GTPases. RAB5 is associated with early endosomes, while RAB7 is involved in the formation of late endosomes, by regulating the fusion of MVBs with lysosomes. On the contrary RAB27 regulates the docking and fusion of MVBs with the plasma membrane (Stenmark, 2009). On the plasma membrane sites soluble N-ethylmaleimide-sensitive factor attachment protein receptors (SNAREs) can promote exosome release (Cai et al., 2007; Piper and Katzmann, 2007; McGough and Vincent, 2016).

Following their release into the intercellular space, exosomes are internalized by target cells by three mechanisms: docking to membrane receptors, fusing directly with the plasma membrane, or following the endocytic pathway (Jiao et al., 2021) thereby contributing to donor cell-mediated biological effects. The effects of exosomes internalization may include epigenetic reprogramming and subsequent phenotypic alterations by activating or inhibiting various signaling pathways, according to the molecular information received from the donor cells (Povero et al., 2015; Ban et al., 2016).

Potential regulation of exosome secretion by mTOR signaling pathway has emerged as a novel research field in liver fibrosis, however, whether it inhibits or induces secretion remains controversial. Exosomes are considered both as an intercellular communication mediator and as an alternative pathway to eliminate excessive and damaged cellular components. It has been demonstrated that exosome release is regulated by mTOR pathway in response to changes in nutrient and growth factors, as well to stress exposure (Zou et al., 2019). Under adverse conditions, which lead to inhibition of mTOR pathway, the release of exosomes seems to act in concert with autophagy, by being both upregulated. This concept is explained regarding exosomes as an alternative process, which functions together with autophagy to coordinate cell waste management and thus ensuring maintenance of cellular homeostasis (Zou et al., 2019). On the other hand, it has been shown opposite results considering the role of mTOR in the release of exosomes (Gao et al., 2020). More specifically, during liver fibrosis, exosomes released by injured hepatocytes and LSECs are enriched in PDGF molecules, which are encapsulated by qHSCs, thereby inducing their activation and migration. Downstream targets of PDGF activate mTOR signaling pathway, thereby leading to upregulation of exosome release from aHSCs, by inhibiting autophagic degradation of MVBs (Gao et al., 2020).

#### Role of Exosomes in Liver Fibrosis

Maintenance of liver homeostasis and functionality requires continuous and highly regulated intercellular communication, which is mainly mediated *via* exosomes (Geerts, 2001; Malarkey et al., 2005). However, studies have explored emerging roles for exosomes in the pathogenesis of liver inflammation, fibrosis, and portal hypertension (Witek et al., 2009). Liver diseases often result in alterations in the number of secreted exosomes and/or in different cargo sorting in exosomes, thus they may differ both quantitively and qualitatively from physiological to disease state (Raposo and Stoorvogel, 2013).

In regards to exosomes, despite being thoroughly studied under pathological conditions, this type of EVs is also secreted in the normal liver by all cell types, contributing to intercellular communication and maintenance of homeostasis (Deng et al., 2017).

Under normal conditions qHSCs secrete exosomes, the cargo of which reflects their metabolic and phenotypic state (Chen et al., 2014; Li et al., 2020). qHSCs are resting cells with vitamin A storing capacity, thus the number of their secreted exosomes is on basal levels. Furthermore, the cargo sorting into qHSCs exosomes is mainly associated with antifibrogenic properties. This cargo may include various components such as transcription factors, miRNAs, and proteins. Considering the transcription factors, it has been indicated that the helix-loop-helix transcription factor Twist 1 could be a potential exosomal cargo secreted by qHSCs, thereby contributing to the maintenance of their quiescent phenotype (Chen et al., 2015). Furthermore, miRNAs, such as miRNA-214 and miRNA-199a-5p, have been shown to be included in quiescent secreted exosomes. The same group has also provided evidence that these miRNAs, with their expression levels being regulated by Twist-1, could lead to suppression of fibrogenic molecules expressed from aHSCs or injured hepatocytes, such as cellular communication network factor 2 (CCN2) (Chen et al., 2015), thereby maintaining a dynamic balance of HSCs quiescent phenotype (Chen et al., 2016). Considering the protein cargo enclosed in exosomes, qHSCs secrete exosomes enriched in protein molecules such as histones and keratins, with this molecular profile being hypothesized to act as an inhibitory regulator of HSCs activation (Li et al., 2020). Thus, the basal number and the cargo of qHSCs secreted exosomes result in maintenance of HSCs homeostasis and inhibition of acquisition of fibrogenic properties.

Chronic liver injury and disruption of the functional properties of liver cells result in fibrotic wound healing process. Activation of HSCs, a dominant phenomenon of liver fibrosis, is highly coordinated by intercellular communication between HSCs and other liver cell types (Hirsova et al., 2016; Cai et al., 2017). Injury stimuli alters the liver microenvironment, thereby affecting both the number and the cargo sorted in exosomes secreted by all hepatic cell types. HSCs, as a central contributor of fibrosis, may act both as an exosome donor and as an exosome recipient cell. Considering HSCs as an exosome recipient cell, it is evident that exosomes secreted by neighboring liver cells promote various genetic and phenotypic alterations when delivered by HSCs, such as myofibroblast-like transdifferentiation, proliferation and migration. Migration specifically, one of the key properties of aHSCs, is highly regulated by LSECs, which secrete exosomes enriched in protein molecules such as sphingosine kinase-1 SK1, thus navigating aHSCs to sites of fibrogenesis (Wang et al., 2015). The fibrotic process is also aggravated by exosomes secreted by LSECs, hepatocytes and KCs, which are enriched in growth factors (GF), such as PDGF. These exosomes inhibit the autophagic process in the aHSCs, contributing in upregulation of exosome secretion, thereby mediating further fibrotic process (Gao et al., 2020).

**FIGURE 2:**
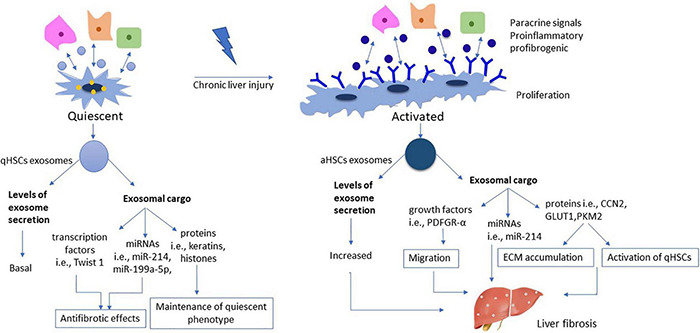
Role of exosomes during HSC activation. The number of exosomes derived from qHSCs is on basal levels, reflecting their metabolic phenotype as resting, vitamin A storing cells. Exosomal cargo includes various transcription factors (i.e., Twist 1) miRNAs (i.e., miR-214, miR-199a-5p) and proteins (i.e., keratins, histones), all being associated with antifibrotic properties and maintaining a dynamic balance in qHSCs phenotype. On the other hand, aHSCs are characterized by increased exosome secretion reflecting the phenotypical transformation into highly proliferative myofibroblastic cells. On this context, the cargo encapsulated in aHSCs exosomes consists of growth factors (i.e., PDGFR-α), miRNAs (i.e., miR-214) and proteins (i.e., CCN2, GLUT1, PKM2), all being associated with further activation of qHSCs and propagation of the fibrotic process.

During the activation process, levels of aHSCs secreted exosomes are upregulated, an alteration which reflects the phenotypic changes from qHSCs to aHSCs, which are highly proliferative, migratory and with elevated energy requirements (Li et al., 2020). Exosomes secreted by aHSCs contain high levels of protein molecules such as CCN2. CCN2 in turn could be delivered by other qHSCs or aHSCs, thereby inducing further expression of CCN2 and aSMA proteins (Charrier et al., 2014) and contributing to the propagation of the fibrotic process. The role of exosomes under normal and pathological conditions appears in Figure 2.

Although the effects of exosomes derived from aHSCs and other parenchymal and non-parenchymal cells on HSCs themselves have been well demonstrated, relatively few studies have elucidated the role of aHSCs as exosome donor cells and the impact of these exosomes on liver microenvironment. During liver fibrosis except for HSCs, both hepatocytes and other non-parenchymal cells undergo genetic and phenotypic modifications, with the intercellular communication *via* exosomes playing crucial role in these changes (Seo and Jeong, 2016). Particularly exosomes derived from aHSCs participate in intercellular communication with other liver cell types such as LSECs. In fact, *in vitro* studies in models of induced activation of HSCs, have shown that aHSCs derived exosomes contain protein molecules, such as hedgehog ligands, which induce genetic modification in LSECs, leading them to an “activated” angiogenic phenotype, thus promoting fibrotic process (Witek et al., 2009). Moreover, further investigations have shown that aHSCs exosomes may also contain various growth factors such as VEGF, which after being incorporated by LSECs promote their angiogenetic phenotype, a characteristic feature of fibrotic LSECs (Thabut and Shah, 2010). Furthermore, other aHSCs exosomal protein molecules include glycolytic markers, such as glucose transporter 1 (GLUT1) and pyruvate kinase M2 (PKM2). These exosomes are transferred not only among neighboring qHSCs but also among other non-parenchymal liver cells, such as KCs and LSECs thus inducing their activation (Wan et al., 2019). A recent study has shown in *in vitro* cultures of aHSCs that aHSCs derived exosomes affect KCs by stimulating cytokine synthesis release and migration (Benbow et al., 2021).

Various *in vitro* and *in vivo* studies have shown that exosome-mediated protein and miRNA transport is a critical mechanism during liver fibrosis. *In vitro* studies have indicated aberrant miRNAs as potential exosomal cargos derived from aHSCs that may regulate hepatic fibrogenesis. However, the results of some studies remain controversial, as it has not been concluded whether some exosomal cargoes are secreted from qHSCs or aHSCs. For example, *in vitro* studies of Ma et al. (2018) have shown that miR-214 is significantly upregulated during HSCs activation process, in contrast to the results of Chen et al. (2015) who found that miR-214 is enriched in qHSCs derived exosomes. Moreover, it has also shown that in mouse model, that levels of miR-214 were upregulated during the progression of liver fibrosis (Ma et al., 2018). *In vivo* studies in induced fibrotic mouse model have revealed that levels of miR-30a were downregulated in aHSCs-exosomes. Normally, miR-30a inhibits autophagy by directly inhibiting Beclin 1, a crucial autophagic associated protein, and increases lipid accumulation in HSCs. Thus, decreased levels of miR-30a in aHSCs exosomes may contribute to further induction of autophagy during the activation process and degradation of lipid droplets, contributing to the transdifferentiation of HSCs (Chen J. et al., 2017; Zheng et al., 2018). Other *in vivo* experiments also supported the observation that exosomes produced by aHSCs promote fibrosis by acting on adjacent qHSCs or other non-parenchymal cells (Charrier et al., 2014; Wan et al., 2019). Considering that platelet-derived growth factor (PDGF)-BB is a key molecule in the process leading to liver fibrosis, it has been reported that PDGF-BB–treated HSCs release PDGF receptor-alpha (PDGFRα)-enriched exosomes, which promote HSC migration and liver fibrosis (Kostallari et al., 2018).

### Crosstalk Between Autophagy and Exosomes- a Coordinated Loop

The importance of autophagy in the maintenance of energetic balance and in cellular quality control in the liver has been well established (Schneider and Cuervo, 2014). Moreover, exosomes have been identified as a significant carrier of intercellular communication between liver cells, which constitute a part of a highly complex and multicellular organ (Kowal et al., 2014; Sung et al., 2018). Therefore, the ability of exosomes to cooperate with autophagy flux for perceiving cellular homeostasis has recently been reported (Gudbergsson and Johnsen, 2019). Both processes may synergically and alternatively act to support cell survival (Baixauli et al., 2014), with these interconnected functional processes being considered as crucial mediators of the hepatic homeostatic process.

The interplay between exosomes and autophagy has been well demonstrated by multiple studies as both autophagic molecules have been implicated in the late endosome distribution and exosome biogenesis and components of the endosomal trafficking machinery have been also associated with the autophagosome maturation (Baixauli et al., 2014).

Stressful stimuli and pathological conditions affect autophagy through the release of exosomes and their cargos. Both exosomal cargo and their origin cell seem to play decisive role in the regulation of autophagy in the recipient cell (Jiang et al., 2018; Jin et al., 2019). On the contrary, autophagy levels may modulate the formation and release of exosomes, demonstrating that these pathways are cross-regulated and both affect the development of various diseases (Fader and Colombo, 2006; Xu et al., 2018). Thus, under both physiological and pathological conditions, the coordination between exosome–autophagy networks serve as a tool to conserve cellular homeostasis *via* the lysosomal degradative pathway and/or secretion of cargo into the extracellular milieu (Baixauli et al., 2014; Salimi et al., 2020).

#### Autophagy Affects Exosomes Biogenesis

##### Molecular Interaction- a Synergistic Relationship Between Exosomes and Autophagy

Emerging evidence suggests direct links between autophagy and exosome biogenesis through intertwined molecular machinery. The autophagy-related proteins (ATG) 5 and 12 (ATG5-ATG12) and the autophagy-related 16 like 1 (ATG16L1) complex, along with LC3-II, have been observed to localize to endosomes and have a curtail role in exosome biogenesis (Florey et al., 2011; Martinez et al., 2015). On this context, a recent report has demonstrated the non-autophagic functions of ATG5, which mediates the dissociation of vacuolar proton pumps (V_1_–V_0_-ATPase) from MVBs, thus preventing the acidification of MVB lumen and allowing fusion with the plasma membrane. Hence the luminal pH of the MVBs plays a key role in controlling whether MVBs undergo lysosomal degradation or plasma membrane fusion. Moreover LC3-II has been demonstrated to be located on the lumen side of MVBs, while intact LC3-II positive exosomes are released in the extracellular environment (Guo et al., 2017). Another study has indicated that ATG12-ATG3, a protein complex required for LC3 lipidation, interacts with Alix, an ESCRT-associated protein, and controls MVB morphology, distribution, and function (Murrow et al., 2015).

**FIGURE 3:**
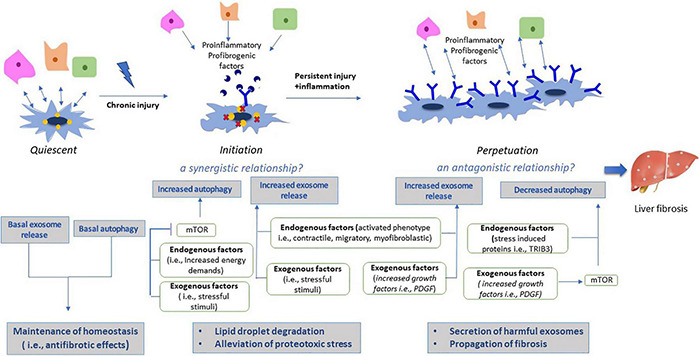
Effects of autophagy and exosomes during HSC activation. Under normal conditions, qHSCs are characterized by basal autophagic levels and exosome release, indicative of their phenotypic characteristics as a resting, vitamin-A storing cell. Both autophagy and exosomal cargo contribute to maintenance of homeostasis by their antifibrotic effects. On the other hand, activation process is divided in two phases; initiation and perpetuation. During initiation phase, liver injury results in increased stress levels, thereby inducing autophagy by mTOR signaling pathway. However, aggregation of harmful substances derived from injured liver cells, exceeds autophagy capacity to degrade them, thus exosome secretion is synergistically upregulated as an alternative pathway to alleviate proteotoxic stress. In addition, activation of HSCs requires high energy levels, thus energy deprivation results in activation of lipophagy and lipid droplet degradation. During perpetuation phase, aHSCs have now been adapted to their new phenotype and micronenvironment, thus they no longer confront energy deprivation, resulting in decreased autophagic levels. Downregulation of autophagy has been potentially associated with induction of harmful secretion of exosomes from aHSCs, thus resulting in propagation of liver fibrosis.

Zou et al. (2019) have examined this synergistic relationship particularly in the liver in an animal model. This research team has shown that induced activation of mTOR signaling pathway inhibited exosome release in liver cells, by downregulation of exosomal markers. Thus, mTOR as an undoubtful inhibitor of autophagy has been demonstrated to regulate concomitantly exosome release in the liver, with this concurrent regulation being considered to play a major role in the orchestration of cellular responses in waste management under adverse conditions (Zou et al., 2019). The synergistic relationship between autophagy and exosomes is displayed in Figure 3.

##### Vesicular Interaction Mechanism- an Antagonist Relationship Between Exosomes and Autophagy

Autophagy levels affect the number of secreted exosomes, as it has been demonstrated that autophagy and exosomes are reciprocally regulated. Inhibition of autophagy lead to increased exosomal secretion, while autophagy induction prevented extracellular release of exosomes (Fader et al., 2008).

Exosomes which are internalized by target cells *via* the endocytic pathway could follow two possible pathways: degradative or secretory (Hessvik and Llorente, 2018). Exosomes which follow the degradative pathway, could either be transported directly to lysosomes for degradation or by forming amphisomes, which will then fuse with the lysosomes (Zheng et al., 2019). Amphisomes are hybrid structures, formed by fusion of autophagosomes with late endosomes/MVBs, which eventually fuse with lysosomes and dissolve internal substances of MVBs such as ILVs (Xu et al., 2018). On the other hand, MVBs could escape degradation pathways and be released by MVB-plasma membrane fusion as exosomes. Therefore, intracellular autophagy levels play a decisive role in the number and fate of exosomes, as increased levels of autophagy inhibit release of exosomes and induce their lysosomal degradation (Fader and Colombo, 2006; Fader et al., 2008; Villarroya-Beltri et al., 2016). On the contrary, during lysosomal or autophagic dysfunction, intracellular autophagic cargo may be released into the extracellular milieu *via* exosomes, which escape from the degradative lysosomal pathway (Ejlerskov et al., 2013; Minakaki et al., 2018).

The vesicular interaction between autophagy and exosomes has also been demonstrated in the liver. It has been shown that during liver fibrosis exosomes released from injured LSECs and Kupffer cells contain high levels of PDGF molecules (Gao et al., 2020). In turn PDGF after internalized by aHSCs, leads to activation of Src homology 2 (SH2)-containing protein tyrosine phosphatase 2 (SHP2), a downstream signaling molecule, which has been shown to suppress REDD1 (regulated in development and DNA damage responses 1), an endogenous inhibitor of mTOR signaling. Thus, activated mTOR signaling lead to increased exosome secretion by attenuating autophagic degradation of late endosomes/MVBs. The enhanced circulation of exosomes released by aHSCs has been associated with further propagation of fibrotic signals (Gao et al., 2020). In addition, it has been reported that increased expression of stress responsive proteins like Tribbles homolog 3 (TRIB3) in injured liver and the consequent dysfunction of the autophagy receptor Sequestosome 1 SQSTM1/p62, have been associated with autophagy impairment and induction of harmful exosomes secretion (Zhang et al., 2020). The antagonistic relationship between autophagy and exosomes is depicted in Figure 3.

#### Activation of Intracellular Autophagy by Exosomes

Intriguingly emerging evidence indicates that exosomes could also affect autophagic levels by encapsulating autophagic proteins as their cargo, such as SQSTM1, LC3 (Hessvik et al., 2016) or autophagy associated mRNAs such as LC3, Beclin1 and ATG7 mRNAs, thus initiating directly autophagy after entering the target cells (Fazeli and Wehman, 2017). In addition, inhibition of ALIX, an ESCRT associated protein, has been reported to decrease also autophagy flux (Murrow et al., 2015), thus indicating a regulatory cross-link between exosome biogenesis and autophagy pathway. Moreover, it has been demonstrated that exosomes could interact with the PI3K/Akt/mTOR or AMPK/mTOR pathways, which are key regulators of autophagy, thus promoting cell survival by reducing immoderate autophagy or induce autophagy, respectively (Liu et al., 2017).

The effect of exosomes on autophagy depends on its parental cells and the conditions that stimulate their production. More specifically, evidence has been provided that exosomes secreted by mesenchymal stem cells can induce moderate autophagy on target cells, thus playing a protective role, while on the other hand exosomes produced by injured cells can transfer harmful substances, induce inappropriate autophagy, and damage normal cells (Zhu et al., 2018). In fact a recent study has demonstrated that exosomes derived from adipose derived mesenchymal stem cells (ADSCs) could improve liver fibrosis by activating autophagy (Zhu et al., 2020), therefore highlighting that these pathways could be considered as an interconnected mechanism underlying liver fibrosis.

## Discussion

Liver constitutes a complex and multicellular organ; thus, maintenance of homeostasis is crucial to ensure proper function. Autophagy plays a key role in the homeostatic process through the degradation of protein aggregates and dysfunctional organelles (Rautou et al., 2010; Codogno and Meijer, 2013; Czaja et al., 2013). Moreover, intercellular communication between HSCs and other parenchymal and non-parenchymal liver cells plays a crucial role in the orchestration of liver responses, thus promoting homeostasis (Sung et al., 2018). On this context, exosomes have recently elucidated as a significant means of cell-to-cell communication (Kowal et al., 2014), therefore both these pathways, autophagy and exosomes seem to closely interact to maintain liver functionality. However, this crossregulation has been recently investigated under pathological conditions, such as liver fibrosis (Gao et al., 2020). Both pathways are significantly affected by injury stimuli; levels of the autophagic flux may be upregulated or downregulated depending on the liver cell type, thereby promoting distortion of the normal hepatic architecture (Rautou et al., 2010; Codogno and Meijer, 2013; Czaja et al., 2013). Moreover, exosomes are characterized by different levels of secretion and different cargo sorting, depending on the underlying conditions that stimulated their secretion and the parental cells (Raposo and Stoorvogel, 2013).

Liver fibrosis, regardless of the etiologic factor, is characterized by cell death of hepatocytes and suppression of liver regeneration, resulting in replacement of normal liver tissue by accumulated ECM (Friedman, 2008b). Concomitantly, injured hepatocytes together with other stressed non-parenchymal cells such as KCs and LSECs secrete exosomes containing inflammatory cytokines and stress-inducing molecules. These exosomes are transferred between qHSCs and prepare them for their activation (Wang et al., 2015). Activation of qHSCs is the most prominent event during the fibrotic process, as aHSCs are the major effector cells for excess matrix deposition (Friedman, 2008a). Studies have demonstrated that transformation of qHSCs from the adipogenic to the myofibroblast profile is mediated through stress-induced autophagy, which has been shown to be strictly regulated by the mTOR signaling pathway (Friedman, 2008b; Wang H. et al., 2019). However, studies in fibrotic liver tissue have shown downregulated levels of stress-induced autophagy (Zhang et al., 2020), with increased exosome containing fibrogenic molecules secreted form aHSCs (Charrier et al., 2014). Considering the above data, we can assume a putative mechanism through which liver fibrosis develops. As already mentioned, increased levels of autophagy play a key role in activating qHSCs, acquiring myofibroblast phenotype. Presumably, the initially increased levels of autophagy could be reduced in the later stages of fibrosis, leading to increased release of fibrogenic exosomes from aHSCs.

Activation of HSCs consists of two distinct phases; initiation and perpetuation (Lee and Friedman, 2011). During initiation induction of mTOR- mediated autophagy in qHSCs occurs in response to incorporation of stress-induced exosomes derived from liver microenvironment and to energy deprivation due to increased metabolic needs of the transdifferentiation process (Wang H. et al., 2019). Under these conditions autophagy levels are upregulated in order to reduce incorporated exosomal toxic substances through lysosomal degradation and to provide enough nutrients necessary for the activation of qHSCs through lipophagy, respectively. The elevated levels of autophagy have been demonstrated in aHSCs, both *in vitro* in human tissue and *ex vivo* in rodent models of liver injury, by a significant increase of autophagic vacuoles, LC3-II levels and autophagic flux (Thoen et al., 2011; Hernández-Gea et al., 2012). However, constant stressful stimuli result eventually in the accumulation of protein aggregates and damaged organelles, which exceed the autophagic capacity of qHSCs to get rid of these molecules. Under these conditions, we assume that concomitant induction of exosome secretion may act as a survival option for the cell in order to alleviate proteotoxic substances by releasing them into the extracellular environment. Thus, at this phase, exosome secretion and autophagy could potentially act synergistically to help qHSCs counter cellular stress (Zou et al., 2019).

On the other hand, once HSCs are activated and become transdifferentiated into myofibroblast-like cells, they probably no longer confront stressful conditions, neither increased energy demands as they have been adapted to their new “normal” situation, and their current activity is to promote ECM accumulation and liver fibrosis (Lotersztajn et al., 2005; Mallat and Lotersztajn, 2013). The downregulated levels of stress induced autophagy have been demonstrated by the activation and enhancement of PI3K/Akt/mTOR signaling pathway in the fibrotic liver (Peng et al., 2017; Lei et al., 2019) thus, confirming that during perpetuation phase stress induced autophagy is no longer triggered, and only basal levels of autophagy continue to exist. Under these conditions, a recent study has shown that downregulation of the autophagic levels result in increased exosome secretion by aHSCs, probably by inhibiting their lysosomal degradation, thereby contributing to the propagation of liver fibrosis. More specifically, it has been demonstrated that liver injury leads to release of PDGF- enriched exosomes from liver microenvironment, such as LSECs and KCs (Gao et al., 2020). These exosomes contain high levels of PDGF molecules. In turn, PDGF binds to its receptor on HSCs and its downstream signaling molecule SHP2 activates indirectly mTOR signaling pathway. Thus, inhibition of autophagy may contribute to increased exosome secretion by attenuating lysosomal degradation of MVBs (Gao et al., 2020). Therefore, we can assume that exosomes and autophagy could be probably reciprocally regulated during the perpetuation phase, as the current activity of aHSCs is the propagation of liver fibrosis by transferring fibrosis inducing molecules toward the liver microenvironment.

The pivotal role of autophagy and exosomes and their multifaceted crossregulation during the different stages of activation process remain a controversial research topic. Up to date, studies have shown that induced autophagy of aHSCs in fibrotic liver seems to act as an inhibitor of the fibrotic process by promoting cell death of aHSCs (Piguet et al., 2014; Zheng et al., 2018). However, they have not yet precisely examined whether this process concerns initiation or perpetuation HSCs and what are the levels of autophagy that are necessary in order to induce cell death of aHSCs and inhibit propagation of hepatic fibrosis. As far as the exosomes are concerned, they are still at a very early stage of research and further studies are necessary to gain further insight in all their possible functions and to understand how their elevated levels of secretion from aHSCs affects the propagation of liver fibrosis during the different stages of activation. In addition to the overlapping molecules and vesicular pathways that have been revealed to link autophagy and exosomes, further investigation is necessary to gain insight in the complete mechanism through which they interact with each other and how this interaction is regulated during the different stages of HSCs activation and fibrotic process. Further studies are needed to examine how this interaction affects both HSCs and other liver parenchymal and non-parenchymal cells and what conditions in the liver microenvironment stimulate the synergistic or antagonistic relationship between autophagy and exosomes.

## Author Contributions

AC conceived and designed the review, studied the literature, wrote the manuscript, and revised the schemes. EM studied the literature, wrote the manuscript, and drew the schemes. AG, GG, and PK studied the literature, revised the manuscript critically for its content and performed modifications. All authors accepted the final version of the review and approved it for publication.

## Conflict of Interest

The authors declare that the research was conducted in the absence of any commercial or financial relationships that could be construed as a potential conflict of interest.

## Publisher’s Note

All claims expressed in this article are solely those of the authors and do not necessarily represent those of their affiliated organizations, or those of the publisher, the editors and the reviewers. Any product that may be evaluated in this article, or claim that may be made by its manufacturer, is not guaranteed or endorsed by the publisher.

## Abbreviations

aHSCs: activated hepatic stellate cells
Akt: Protein kinase B (PKB)
Alix: apoptosis-linked gene-2 interacting protein X
CCN2: communication network factor 2
ECM: extracellular matrix
ESCRT: endosomal sorting complex required for transport
ET-1: endothelin-1
EVs: extracellular vesicles
HSCs: Hepatic stellate cells
ILVs: Intraluminal vesicles
KCs: Kupffer cells
LSECs: Liver sinusoidal endothelial cells
MMPs: matrix metalloproteinases
mTOR: mammalian Target of Rapamycin
MVBs: Multivesicular bodies
PDFGR- β: platelet-derived growth factor receptor beta
PDGF: platelet-derived growth factor
PI3K: Phosphoinositide 3-kinase
PPAR γ: peroxisome proliferator-activated receptor gamma
qHSCs: quiescent hepatic stellate cells
SHP2: Src homology 2 (SH2)-containing protein tyrosine phosphatase 2
SNAREs: N-ethylmaleimide -sensitive factor attachment protein receptors
TGF- β: transforming growth factor beta
TIMPs: tissue inhibitors of metalloproteinases
α SMA: α-Smooth muscle actin.
